# β-elemene alleviates airway stenosis via the ILK/Akt pathway modulated by MIR143HG sponging miR-1275

**DOI:** 10.1186/s11658-021-00261-0

**Published:** 2021-06-12

**Authors:** Guoying Zhang, Cheng Xue, Yiming Zeng

**Affiliations:** 1 Department of Pulmonary and Critical Care Medicine, Respiratory Medicine Center of Fujian Province, The Second Affiliated Hospital of Fujian Medical University, Zhongshan North Road No.34, Licheng District, Quanzhou, Fujian China; 2 Department of Pulmonary and Critical Care Medicine, Quanzhou First Hospital of Fujian Medical University, Quanzhou, Fujian China; 3 Department of Pulmonary and Critical Care Medicine, The First Affiliated Hospital of Xiamen University, Xiamen, Fujian China

**Keywords:** Tracheal stenosis, Fibroblast, β-elemene, CeRNA, ILK/Akt pathway

## Abstract

**Background:**

We have previously found that β-elemene could inhibit the viability of airway granulation fibroblasts and prevent airway hyperplastic stenosis. This study aimed to elucidate the underlying mechanism and protective efficacy of β-elemene in vitro and in vivo.

**Methods:**

Microarray and bioinformatic analysis were used to identify altered pathways related to cell viability in a β-elemene-treated primary cell model and to construct a β-elemene-altered ceRNA network modulating the target pathway*.* Loss of function and gain of function approaches were performed to examine the role of the ceRNA axis in β-elemene's regulation of the target pathway and cell viability. Additionally, in a β-elemene-treated rabbit model of airway stenosis, endoscopic and histological examinations were used to evaluate its therapeutic efficacy and further verify its mechanism of action.

**Results:**

The hyperactive ILK/Akt pathway and dysregulated LncRNA-MIR143HG, which acted as a miR-1275 ceRNA to modulate ILK expression, were suppressed in β-elemene-treated airway granulation fibroblasts; β-elemene suppressed the ILK/Akt pathway via the MIR143HG/miR-1275/ILK axis. Additionally, the cell cycle and apoptotic phenotypes of granulation fibroblasts were altered, consistent with ILK/Akt pathway activity. In vivo application of β-elemene attenuated airway granulation hyperplasia and alleviated scar stricture, and histological detections suggested that β-elemene's effects on the MIR143HG/miR-1275/ILK axis and ILK/Akt pathway were in line with in vitro findings.

**Conclusions:**

MIR143HG and ILK may act as ceRNA to sponge miR-1275. The MIR143HG/miR-1275/ILK axis mediates β-elemene-induced cell cycle arrest and apoptosis of airway granulation fibroblasts by modulating the ILK/Akt pathway, thereby inhibiting airway granulation proliferation and ultimately alleviating airway stenosis.

**Supplementary Information:**

The online version contains supplementary material available at 10.1186/s11658-021-00261-0.

## Background

Benign airway stenosis is caused by non-malignant factors, including endotracheal intubation, tuberculosis, tracheotomy, systemic diseases, and tracheal anastomosis. This condition results in varying degrees of dyspnea or even death by asphyxia [1, 2]. Remarkable advances in interventional pulmonology, and transbronchoscopic intervention (such as balloon dilation, laser or electrotomy, and stent placement) have made it a major method of treating benign airway stenosis. However, long-term benefits are limited [3–5]. In some cases, the interventional therapeutic bronchoscopy will cause secondary damage to the airway structures, and granulation tissue hyperplasia and scarring during the wound healing will make the stenosis relapse. It may cause repeat bronchoscopy, resulting in a vicious cycle of "stenosis–intervention with impairment–restenosis after repair" [6]. Fibroblasts, a major component of granulation tissue, play a dominant role in granulation tissue hyperplasia and scarring [7, 8]. Human airway granulation pathological features resemble those of hypertrophic scarring. Mounting evidence has associated hypertrophic scarring with abnormal fibroblast activity in the wound bed [9]. Abnormal fibroblasts exhibit greater proliferation potential and resistance to apoptosis, worsening severe fibrosis responses.

β-Elemene is isolated from Rhizoma Curcumae and is widely used as an anticancer adjuvant as it enhances the effects of many anticancer drugs with minimal toxicity [10]. In addition, many studies have shown that β-elemene also has an anti-fibrosis effect. It was found to be effective in inhibiting the viability of various fibroblast‐like cells, including hepatic stellate cells [11], human rheumatoid arthritis fibroblast-like synoviocytes [12], and human glioblastoma cells [13], thereby retarding the progression of relevant diseases. We have previously reported β-elemene’s anti-proliferation and pro-apoptosis effects on primary human airway granulation fibroblasts (PHAGF) in vitro and in vivo [14]. However, the underlying mechanisms remain elusive.

Recent studies indicate that non-coding RNAs (ncRNAs) modulate almost every aspect of gene expression and pathway activation [15]. Among them, long non-coding RNAs (lncRNAs), macro ncRNAs with a length of over 200 nucleotides, have been implicated in gene regulation at epigenetic, transcriptional, and post-transcriptional levels [16]. One of the important mechanisms by which they function is that they could act as competing endogenous RNA (ceRNA) for miRNAs [17, 18]; hence they regulate multiple biological processes, such as apoptosis, cell cycle progression and invasion, and influence the pathogenesis of various diseases [19–21]. LncRNAs have emerged as disease biomarkers and therapeutic targets [22–24]. It has also been reported that β-elemene suppresses esophageal cancer cell proliferation via lncRNA CDKN2B-AS1 [25]. It is therefore plausible that lncRNAs influence β-elemene effects on human airway granulation fibroblasts. Here, we sought to uncover differential lncRNAs and mRNAs expressed in human airway granulation fibroblasts after β-elemene treatment by high-throughput transcriptome microarray combined with bioinformatic approaches to examine the mechanism underlying β-elemene's suppression of human airway granulation fibroblast viability and to determine β-elemene's efficacy and further validate the mechanism of its action in an animal model of tracheal stenosis, thus providing a theoretical basis and experimental evidence for the clinical application of β-elemene in the treatment of benign airway stenosis and a rationale for identification of promising pharmacotherapy.

## Materials and methods

### Cell culture and treatment

Primary human airway fibroblasts (PHAF) were cultured from normal airway tissues after surgical extraction from a lung cancer patient. Primary human airway granulation fibroblasts (PHAGF) were cultured from airway hyperplastic granulation tissues resulting from prolonged intubation. The hyperplastic granulation tissues were collected during routine bronchoscopy. These cell populations were established and propagated as reported before [26]. Cells were cultured in DMEM (HyClone, Cat# SH30243.01) supplemented with 20% FBS (HyClone, Cat# SH30070.03), at 37 °C, 5% CO_2_. They were passaged every 3 days and passages 3–7 were used in experiments. HEK293T was obtained from the BeNa culture collection (Beijing, China). In this study, cells were treated with β-elemene (Sigma-Aldrich, Cat# SJ05001280278, CAS# 515-13-9), QLT0267 (QLT Inc., Cat# BCP25954, CAS# 866409-68-9) or transfected with oligonucleotides and plasmids individually or in a specified combination for 48 h. This is shown in detail in the figure legends. They were further used for cell proliferation assay, microarray analysis, RT-qPCR, western blotting, and flow cytometric analysis of the cell cycle and apoptosis.

### Determination of β-elemene concentration for assays

We have previously found that β-elemene acts in a concentration-dependent rather than time-dependent manner [27]. To select the appropriate β-elemene dosage, 5.0 × 10^3^ PHAGF cells/well were seeded onto 96-well plates and treated with β-elemene (analytically pure, Sigma-Aldrich, USA) at 0, 10, 30, 60, 120, 240, 360, 480, and 1000 µg/mL for 48 h (β-elemene was dissolved in DMSO and diluted to the desired concentration with the culture medium). Then the inhibition rate of cell proliferation was measured by CCK-8 assay. The half maximal inhibitory concentration (IC50) of β-elemene against PHAGF at 48 h of treatment was calculated from an experimentally derived dose response curve for each concentration using GraphPad Prism version 8.0 (GraphPad Software, CA). The IC50 dosage was used in subsequent treatments. Assays were done in triplicate.

### Cell counting kit-8 (CCK-8) assay

Cell proliferation was assessed using a CCK-8 kit (Beyotime, Cat# C0041) according to the operating manual. In brief, cultured fibroblasts were treated with 0.25% trypsin–EDTA (Gibco, Cat# 25300–054) to prepare a single cell suspension, 3,000 cells were then plated into 96‐well plates. After culture for 1, 2, 3, and 4 days, 10 µl of CCK-8 reagent was added to each well and incubated for 1 h at 37 °C. Finally, the absorbance of OD at 450 nm was measured with an automatic micro-plate reader (Bio-Rad, Hercules, CA, USA).

### Microarray analysis and ceRNA network construction

Three human airway granulation fibroblast samples were cultured on 6-well plates and treated with 1% DMSO (for negative control) or 160 µg/mL β-elemene for 48 h. Microarray analysis was carried out using Arraystar Human lncRNA + mRNA array V.4.0 (Arraystar, Rockville, MD, USA) and performed by Kangchen Biotech Co., Ltd. (Shanghai, China). The main steps were RNA extraction, fluorescein-labeled probe preparation, chip hybridization, image acquisition and data analysis. In brief, sample labeling and array hybridization were performed according to the Agilent One-Color Microarray-Based Gene Expression Analysis protocol. Agilent Feature Extraction software (version 11.0.1.1) was used to analyze acquired array images. Quantile normalization and subsequent data processing were performed using the GeneSpring GX v12.1 software package (Agilent Technologies). The raw and processed microarray data have been submitted to the Gene Expression Omnibus (accession number GSE155297). Differentially expressed lncRNAs and mRNAs were filtered using the criteria: log_2_fold change > 1 or log_2_fold change ≤1 and adjusted *p*≤ 0.05. The differentially expressed mRNAs were then subjected to KEGG pathway analysis to elucidate significantly enriched pathways [28], and ggplot2 was used to generate dotplots for data visualization (adjusted *p* ≤0.05) [29]. False discovery rate (FDR) < 0.25 was considered significant for enriched biological pathways.

To create a putative dysregulated lncRNA‐mediated competing endogenous RNA (ceRNA) network, a regulatory network of lncRNA-miRNA-mRNA interactions based on potential ceRNA triples sourced from miRBase (Sanger miRBase Release 22.1; http://www.mirbase.org) was established [30]. The ceRNA network was visualized in Cytoscape 3.8 [31].

### Transfection

miR‐1275 mimics, negative control mimics (NC-mimics), miR‐1275 inhibitors, negative control inhibitors (NC-inhibitors), pcDNA3.1 which was applied to overexpress MIR143HG or ILK, and negative control-pcDNA3.1 (pcDNA3.1-NC) were obtained from GenePharma (GenePharma Co., Ltd., Shanghai, China). A transfection experiment was conducted when the cell confluence reached approximately 70%. Complete medium without antibiotics was used to culture the cells at least 24 h prior to transfection. The cells were washed with PBS and then transiently transfected with the above oligonucleotides, plasmids or the mix using Lipofectamine 3000 reagent (Invitrogen, Cat# L3000015) as planned according to the manufacturer's protocol. After incubation for 6 h in a cell incubator, the original medium was replaced. The cells were collected 48 h after transfection for the following experiments. The target sequences used were as follows: 5′-GACAGCCUCUCCCCCAC-3′ for miR-1275 inhibitors; 5′-GUGGGGGAGAGGCUGUC-3′ for miR-1275 mimics; 5′-UUCUCCGAACGUGUCACGUTT-3′ for negative control. The sequence information of NC inhibitors was not provided by the GenePharma Company. The final concentrations of miRNAs or plasmids used in this study were as follows: pcDNA3.1-ILK/pcDNA3.1-NC 50 nM/mL, pcDNA3.1-MIR143HG/pcDNA3.1-NC 20 nM/mL, miR-1275 mimics/NC-mimics 30 nM/mL, and miR-1275 inhibitors/NC-inhibitors 150 nM/mL.

### Luciferase reporter assay

miR-1275 binding sites on MIR143HG or ILK 3ʹ-untranslated regions (UTR) were analyzed on miRbase online. The ILK-3ʹ-UTR sequence or MIR143HG cDNA containing predicted miR-1275 binding sites, or their mutated sequence (ILK-mut, MIR143HG-mut), was then ligated to pmirGLO firefly luciferase plasmid (Promega, Cat# E1330), respectively, and the MIR143HG-mut was cloned into a pcDNA3.1 plasmid. The above plasmids were constructed and identified by GenePharma Company. Next, HEK-293 T cells were cultured on 24-well plates and co-transfected with the above plasmids, miR-1275 mimics, NC-mimics, pcDNA3.1-MIR143HG-mut or pcDNA3.1-NC in a specified combination together with Renilla luciferase plasmid (pRL-TK) using Lipofectamine 3000 reagent (Invitrogen, Cat# L3000015). Luciferase activity was measured 48 h after transfection using the Dual-Glo luciferase assay system (Promega, Cat# E2920) and a MicroLumatPlus LB96V luminometer (Berthold, USA) following the protocols of the manufacturer. Firefly luciferase activity was normalized relative to Renilla luciferase activity.

### RT-qPCR analysis

The cells cultured in 6-well plates were used for RT-qPCR analysis. Total RNAs of cells were isolated using TRIzol reagent (TaKaRa, Cat# 9109) and measured using a NanoDrop 2000 (Thermo Fisher Scientific, Waltham, Massachusetts, U.S.). Reverse transcript miRNA was obtained using the One Step PrimeScript miRNA cDNA Synthesis Kit (TaKaRa, Cat# D350A). Reverse transcript lncRNA or mRNA was carried out using the PrimeScript RT reagent kit (TaKaRa, Cat# RR037A) according to the manufacturer’s instructions. SYBR premix ExTaq reagent kit II (TaKaRa, Cat# RR820A) was utilized to conduct the RT-qPCR reaction on the ABI PRISM 7500 sequence detection system (Applied Biosystems, USA). The relative expression levels of lncRNAs and mRNAs were normalized relative to GAPDH while the relative expression levels of miRNAs were normalized relative to U6 and calculated using the − 2^ΔΔ^Ct method. The primers are shown in Table 1.

**Table 1:** Primers used for RT-qPCR

Primer	Forward sequence (5′–3′)	Reverse sequence (5′–3′)
miR-1275	CGTGGGGGAGAGGCTGTC	Involved in the kit
U6	CTCGCTTCGGCAGCACATA	AACGATTCACGAATTTGCGT
MIR143HG	AGAAGCAAGAACTCTGGAGA	TCTGCCTCTGTGTTCCCCATG
ILK	ATGGAACCCTGAACAAACACT	AGCACATTTGGATGCGAGAAA
Akt	GGACAACCGCCATCCAGACT	GCCAGGGACACCTCCATCTC
Bcl-2	TTCTTTGAGTTCGGTGGGGTC	TGCATATTTGTTTGGGGCAGG
Cyclin D1	GTGCATCTACACCGACAACTCCA	TGAGCTTGTTCACCAGGAGCA
GAPDH	CGGAGTCAACGGATTTGGTCGTAT	AGCCTTCTCCATGGTGGTGAAGAC

### Western blotting

Cell lysis and Western blotting were performed as we previously described [27]. Antibodies used in this study were as listed below: anti-ILK (1:800, Affinity, Cat# DF6141, RRID: AB_2838108), anti-Akt (1:1,000, Cell Signaling Technology, Cat# 9272, RRID: AB_329827), anti-Phospho-Akt (Ser473) (p-Akt) (1:2,000, Cell Signaling Technology, Cat# 4060S, RRID: AB_2315049), anti-Cyclin D1 (1:1,000, Cell Signaling Technology, Cat# 55506, RRID: AB_2827374), and anti-Bcl-2 (1:1000, Abcam, Cat# ab32124, RRID: AB_725644), and anti-GAPDH (1:10,000, Abcam, Cat# ab181602, RRID: AB_2630358), anti-rabbit IgG HRP‐conjugated secondary antibody (1:10,000, Abcam, Cat# ab97051, RRID: AB_10679369). Data analysis was conducted using ImageJ software ((ImageJ V1.53b, NIH, USA) and the GAPDH protein was chosen for an internal control. The experiment was repeated at least three times.

### Flow cytometric analysis of cell cycle and apoptosis

Cell cycle analysis was performed using a cell cycle staining kit (Beyotime, Cat# C1052) following the manufacturer’s instructions. Cells were fixed with 70% cold ethanol for 3 h, rinsed using PBS, and then labeled with propidium iodide in the presence of RNase A for 30 min in the dark. Flow cytometry (Beckman Coulter FC500, USA) and FlowJo software (version 7.6.1) were used to determine the percentages of cells in G0/G1, S, and G2/M phases. Apoptosis was analyzed using an Annexin V-FITC/PI apoptosis kit (Beyotime, Cat# C1062M) according to the manufacturer’s instruction. Treated cells were harvested and rinsed twice with cold PBS before staining with 5 μL of Annexin V‐FITC and 5 μL of propidium iodide (PI). They were then incubated in the dark for 15 min at room temperature before analysis by flow cytometry (Beckman Coulter FC500, USA). The PHAGF apoptotic rate was determined by the proportion of Annexin V-FITC positive cells (early apoptosis) plus Annexin V-FITC/PI positive cells (late apoptosis). Cells negative for Annexin V-FITC and PI were considered live cells. Assays were done in triplicate.

### In vivo study on rabbit model of tracheal stenosis

Thirty-five male New Zealand white rabbits (SLAC Laboratory Animal Co., Ltd, Shanghai, China), weighing 4.0–5.0 kg (8 months of age), were used to create New Zealand rabbit models of tracheal stenosis using the modified method we previously established [32]. In the granulation hyperplasia stage (1 week after modeling), modeling rabbits were randomly divided into the NC group and β-elemene group (8 per group). Local injection of tracheal granulation tissue was performed via a bronchoscope (Teslong-NTS300, China): 0.1 mL of 160 μg/mL β-elemene (β-elemene group) or 0.1 mL of 1% DMSO (NC group) was injected into the tracheal granulation at 0, 3, 6 and 9 o 'clock points, respectively.

One week after drug intervention (2weeks after modeling), when modelling rabbits were in the granulation hyperplasia stage, 4 rabbits were randomly selected from each  group and endoscopy was used to compare the tracheal stenosis rate between the two groups. Then the rabbits were sacrificed to extract their tracheal granulation. The tracheal granulations were then divided into four portions. Some of the tracheal granulations were made into 8 μm cryostat sections to detect Ki67 by immunofluorescent staining and for detecting apoptotic cells by TUNEL assay; some of the tracheal granulations were fixed with 4% paraformaldehyde for 24 h, then routine 5 μm paraffin-embedded sections were made. Either H&E staining for pathological assessment or IHC staining to detect the expression of ILK, Akt, p-Akt, cyclin D1 and Bcl-2 was performed. Some of the tracheal granulations were used to extract total RNAs for detecting the expression of MIR143HG, miR-1275, ILK, Akt, cyclin D1 and Bcl-2 by RT-qPCR (the same protocol as described above and the same primers as in Table 1 were used); some of them were to extract proteins for detecting ILK, Akt, p-Akt, cyclin D1 and Bcl-2 expression using Western blotting (the same protocol as described above). At the scar’s mature stage (4 weeks after modeling), the final stenosis rate of the remaining experimental rabbits was measured endoscopically, then their stenotic tracheal segments were dissected for pathological examination through H&E staining.

The methods of endoscopic measurement of stenosis rate, stenotic tracheal segment sampling, fixation, paraffin embedding and H&E staining were the same as those described in our prior work [32]. Primary antibodies used for Western blotting of the tissue protein were: Mouse anti-ILK (1:500, Santa Cruz Biotechnology, Cat# sc-20019 RRID: AB_627807), Mouse anti-Akt (1:1000, Cell Signaling Technology, Cat#2920 RRID: AB_1147620), Mouse anti-Phospho-Akt (Ser473) (1:1000, Cell Signaling Technology, Cat# 4051 RRID: AB_331158), Mouse anti-cyclin D1 (1:1000, Cell Signaling Technology, Cat# 2906 RRID: AB_2070400), Mouse anti-Bcl-2 (1:1000, Cell Signaling Technology, Cat#15071 RRID: AB_2744528). The secondary antibody used was HRP-conjugated goat anti-Mouse IgG (1:2000, Abcam, cat# ab97040, RRID: AB_10698223).

### Immunohistochemistry and immunofluorescent staining

After dewaxing, rehydration, antigen retrieval and blocking nonspecific antigens with 10% goat serum, the 5 μm paraffin sections were incubated with primary antibodies overnight at 4 ℃. For performing immunofluorescent staining, the sections were then incubated with fluorescein conjugated secondary antibody in the dark for 1 h at room temperature, followed by staining with DAPI (0.1 μg/mL, Beyotime, Cat# C1002) in the dark for 10 min to counterstain the nucleus, and images were finally captured using a fluorescence microscope (DMi8, Leica, Germany) after coating the slide with anti-quenching and sealing agents. To perform immunohistochemistry staining, the sections were then incubated with HRP-conjugated secondary antibody for 1 h at room temperature followed by DAB (Main Biotech, Cat# DAB-4032, China) staining in accordance with the kit instructions. Subsequently, the nuclei were counterstained with hematology. Images were finally obtained under an optical microscope (Eclipse Ti2, Nikon). Primary antibodies used for immunohistochemical staining were as listed below: Mouse anti-ILK (1:100, Santa Cruz Biotechnology, Cat# sc-20019, RRID:AB_627807), Mouse anti-Akt (1:200, Cell Signaling Technology, Cat#2920, RRID:AB_1147620), Mouse anti-Phospho-Akt (Ser473) (1:100, Cell Signaling Technology, Cat# 4051, RRID:AB_331158), Mouse anti-cyclin D1 (1:500, Cell Signaling Technology, Cat# 2926, RRID:AB_2070400), Mouse anti-Bcl-2 (1:250, Cell Signaling Technology, Cat#15071, RRID:AB_2744528), Mouser anti-ki67 (1:100, Nevus, Cat# nbp2-22112). The HRP-conjugated secondary antibody used was HRP-conjugated goat anti-Mouse IgG (1:5000, Abcam, Cat# ab97040, RRID: AB_10698223). The fluorescein conjugated secondary antibody used was Alex Floor 488 conjugated goat anti-Mouse IgG (1:1000, Thermo Scientific, Cat# A-11029 RRID: AB_10698223).

### TUNEL (terminal dUTP nick-end labeling) assay

8 μm cryostat sections were analyzed using the One Step TUNEL Apoptosis Assay Kit (meilunbio, Cat# MA0223-1, China). The assay was performed according to the manufacturer’s instructions, followed by counterstaining with DAPI (0.1 μg/mL, Beyotime, Cat# C1002). Apoptotic cells containing fractured DNA labeled with FITC-12-dUTP were photographed under a fluorescence microscope (DMi8, Leica, Germany).

### Statistical analysis

Data analysis and presentation were performed using GraphPad Prism V. 8.0. All assays were done three times. Statistical differences between groups were examined by Student’s *t*-test. Data are shown as mean ± SD of at least 3 independent experiments. *P* < 0.05 indicates statistical significance.

## Results

### β-Elemene-affected ceRNA axis identified via microarray and bioinformatics analysis

**Fig. 1:**
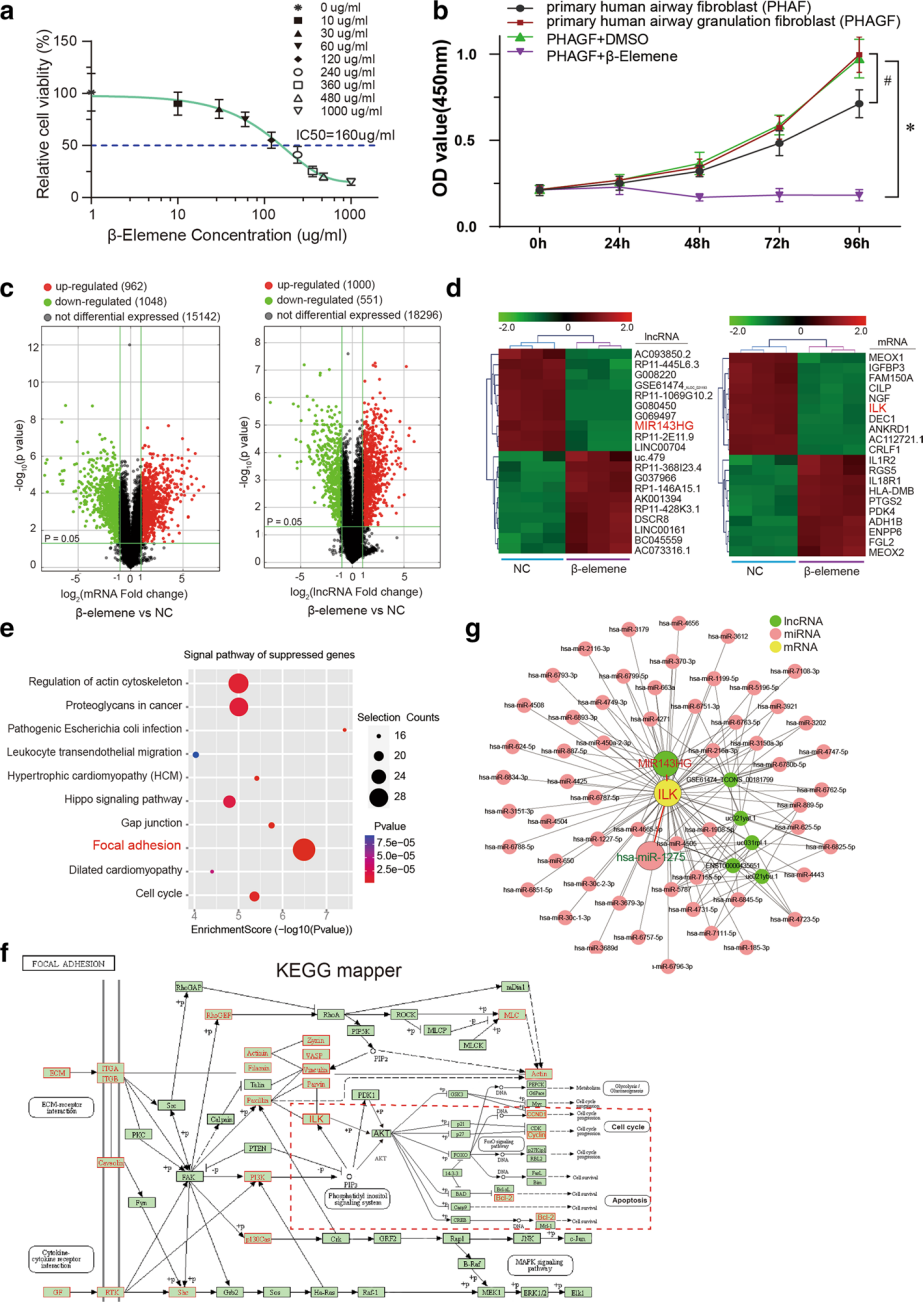
β-Elemene-affected ceRNA axis identified via microarray and bioinformatics analysis **a** Determination of the IC50 of β-elemene in primary human airway granulation fibroblasts (PHAGF) used in subsequent experiments including microarray analysis. **b** CCK‐8 assay-detection of the proliferation of primary human airway fibroblasts (PHAF), PHAGF and β-elemene-treated PHAGF. ^*^P < 0.05, ^#^*p* < 0.05. **c** A volcano plot was generated to show the differential expression distribution via microarray analysis. **d** Heatmap of the top 10 differentially expressed lncRNAs and mRNAs in PHAGF after β-elemene treatment. Green indicates down-regulation, red indicates up-regulation. **e** A dotplot depicts the top 10 suppressed pathways with the highest enrichment scores identified by KEGG pathway enrichment analysis in β-elemene-treated PHAGF. **f** Differentially expressed mRNAs were mapped to the focal adhesion pathway network via KEGG mapper tools. Red solid-line box represents down-regulated gene; large red dashed box indicates the ILK/Akt sub-pathway. **g** Visualization of ILK-centered ceRNA regulatory network constructed from bioinformatic database

PHAGF was treated with β-elemene for 48 h, and the IC50 of β-elemene was 160 μg/ mL, which was calculated by the inhibition rate curve of cell proliferation determined by CCK-8; it was then used as the optimal intervention concentration of β-elemene in subsequent studies (Fig. 1a). Additionally, CCK-8 assay revealed higher proliferation by PHAGF relative to PHAF, and 160 μg/mL β-elemene significantly suppressed PHAGF proliferation (Fig. 1b). In order to explore the possible mechanism of β-elemene acting on PHAGF, we compared the lncRNA and mRNA expression profiles of three PHAGF samples treated with 160 μg/mL β-elemene (β-elemene group) or 1% DMSO (negative control group) using high-throughput microarray analysis. With |log_2_fold change|> 1 and adjusted *p* < 0.05 as the screening criteria, 962 mRNAs and 1000 lncRNAs were up-regulated while 1048 mRNAs and 551 lncRNAs were down-regulated in the β-elemene group, compared with the NC group (Fig. 1c). The differentially expressed mRNAs and lncRNAs with |fold change| ranking in the top 10 were visualized on a heatmap, in which lncRNA-MIR143HG and mRNA-ILK were found significantly down-regulated (Fig. 1d). Next, based on false discovery rate (FDR) < 0.25, KEGG pathway enrichment analysis of differentially expressed mRNAs was done. Visualization of the top 10 suppressed pathways with the highest enrichment score highlighted the focal adhesion signaling pathway (Fig. 1e), meaning that a relatively large number of genes in this pathway were down-regulated. Moreover, the screened suppressed mRNAs mapping to the focal adhesion pathway revealed that ILK and its downstream genes, the cell cycle related gene cyclin D1 and the anti-apoptosis gene Bcl-2 (both of which can be regulated by ILK phosphorylating Akt Ser-473), were enriched in the ILK/Akt sub-pathway (Fig. 1f). Presuming that the ILK/Akt pathway is the target pathway of β-elemene, we constructed a ceRNA network centered on ILK, the hub gene of the ILK/Akt pathway, based on the screened differentially expressed lncRNAs that were positively correlated with ILK and predicted to have at least one common binding miRNA with ILK by miRBase (www.mirbase.org), an online database predicting sequences that interact with miRNAs (Fig. 1g). In this ceRNA network regulating the ILK/Akt pathway, the above significantly down-regulated lncRNA MIR143HG and the predicted miRNA hsa_miR-1275 were selected for further analysis, because both of them were reported to be implicated in the modulation of cell proliferation, and MIR143HG could act as a "sponge" to absorb miR-1275 and hinder its function [33–35].

### β-Elemene inhibited the hyperactive ILK/Akt pathway in PHAGF and PHAGF's cell viability through ILK

**Fig. 2:**
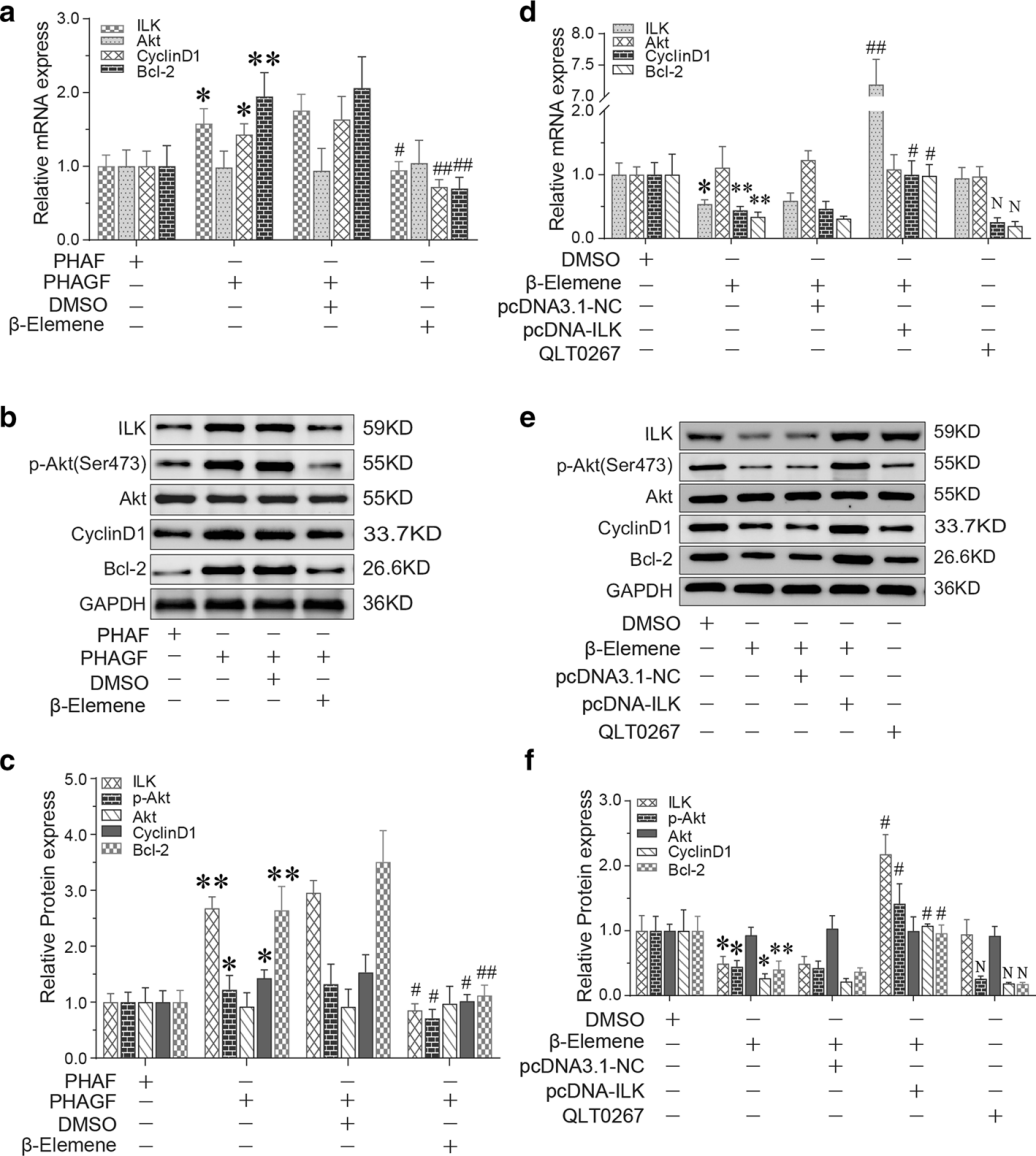
β-Elemene inhibited hyperactive ILK/Akt pathway in PHAGF through ILK. **a** RT-qPCR analysis of the ILK/Akt pathway-related mRNA expression in PHAF, PHAGF and β-elemene-treated PHAGF; **b**, **c** Western blotting analysis of the ILK/Akt pathway-related proteins’ expression in PHAF, PHAGF and β-elemene-treated PHAGF; ^*^P < 0.05, ^**^P < 0.01, versus the PHAF group; ^#^*P* < 0.05, ^##^*P* < 0.01, versus the PHAGF + DMSO group (solvent control). **d** RT-qPCR detection of the transfection efficiency of pcDNA3.1-ILK and to detect the effects of pcDNA3.1-ILK or the pathway inhibitor QLT0267 on expression of ILK/Akt pathway-related mRNAs; **e**, **f** Western blotting detection of the ILK/Akt pathway-related protein expression after ILK over-expression or adding the pathway inhibitor QLT0267 in the presence of β-elemene. ^*^P < 0.05, ^**^P < 0.01, versus the PHAGF + DMSO group; ^#^*P* < 0.05, ^##^*P* < 0.01, versus the β-elemene + pcDNA3.1-NC group; N = no significance, versus the β-elemene group

**Fig. 3:**
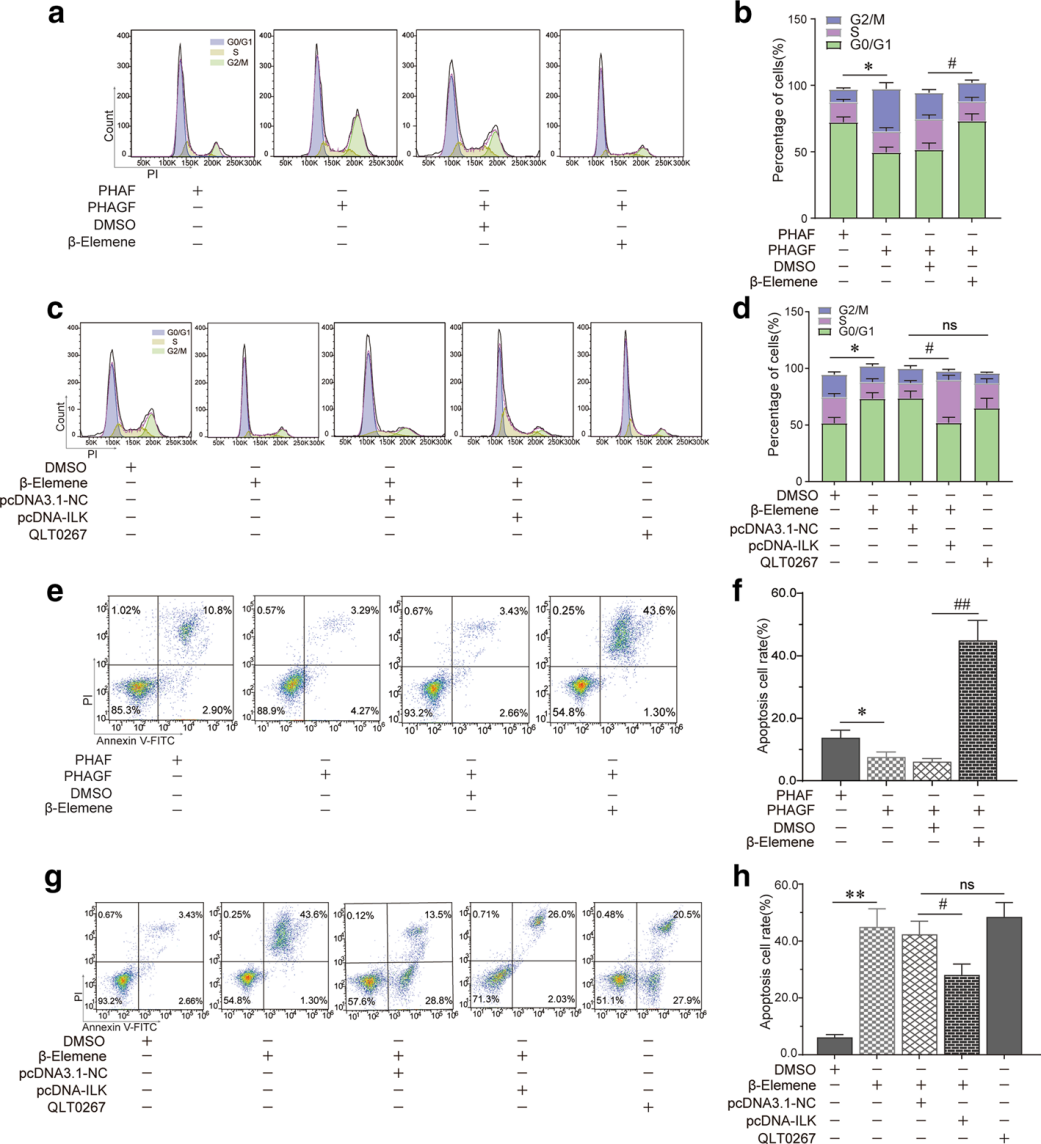
β-Elemene induced PHAGF G0/G1-phase arrest and apoptosis by suppressing ILK/Akt pathway. **a**–**d** Cell cycle distributions in PHAF, PHAGF and PHAGF under indicated treatments were analyzed by flow cytometry, and their corresponding statistical graphs were presented (comparing the rate of cell-cycle arrest in G0/G1-phase). **e**–**h** Apoptosis in PHAF, PHAGF and PHAGF under indicated treatments were analyzed by flow cytometry, and their corresponding statistical graphs were presented (comparing the relative amount of the early apoptosis plus late apoptosis). ^*^P < 0.05; ^**^P < 0.01; ^#^*P* < 0.05; ^##^*P* < 0.01; *ns* not significant

To determine whether the hyperactive ILK/Akt pathway caused the hyperactivity of PHAGF, and whether β-elemene suppressed PHAGF viability by down-regulating this pathway, firstly, we compared the activity of the ILK/Akt pathway and cell cycle progression or cell apoptosis phenotype between PHAGF and PHAF. RT-qPCR and Western blotting detection showed that compared with PHAF, the expression or activity of ILK, Akt, Bcl-2 and cyclin D1 in the ILK/Akt pathway was significantly increased in PHAGF, but was decreased after β-elemene intervention (Fig. 2a–c). Flow cytometry analysis also showed that PHAGF had less cell cycle arrest in G0/G1 phase and a lower cell apoptosis rate than PHAF, and that after β-elemene intervention, PHAGF could return to a normal GO/G1 phase distribution and apoptosis rate like PHAF (Fig. 3a, b, e, f). To confirm ILK mediated β-elemene's regulatory effect on the ILK/Akt pathway and cell cycle or apoptosis, we transfected pcDNA3.1-ILK into PHAGF under treatment with β-elemene; we found that over-expression of ILK rescued the suppressive effect of β-elemene on the ILK/Akt pathway, as RT-qPCR and Western blotting analysis showed that the expression or activity of ILK downstream molecules Akt, Bcl-2 or cyclin D1 in the ILK/Akt pathway were restored (Fig. 2d–f), and over-expressed ILK restored PHAGF viability, as flow cytometry analysis revealed that PHAGF recovered to low G0/G1 phase arrest and a low apoptosis rate (Fig. 3c, d, g, h). Additionally, RT-qPCR and Western blotting analysis demonstrated that when the ILK inhibitor OLT-0267 was added to the PHAGF, it exerted similar effects on the activity of Akt, Bcl-2 and cyclin D1 as β-elemene did (Fig. 2d–f), while flow cytometry analysis revealed that when the ILK inhibitor OLT-0267 was added to the PHAGF medium, it also caused similar effects on the cell cycle arrest and apoptosis as those of β-elemene (Fig. 3c, d, g, h). These results suggest that the hyperactive ILK/Akt pathway in PHAGF leads to hyperfunction of PHAGF and that β-elemene is responsible for inhibiting the ILK/Akt pathway by down-regulating ILK, thereby promoting cell cycle arrest and apoptosis of PHAGF.

### MIR143HG acted as a ceRNA for miR-1275 to promote ILK expression

**Fig. 4:**
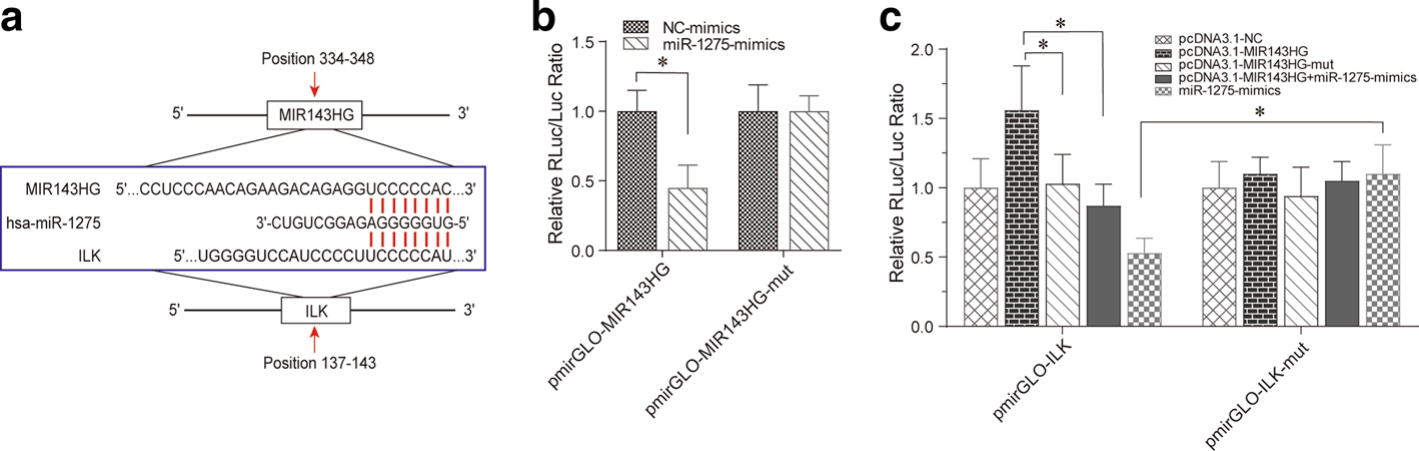
MIR143HG acted as a ceRNA for miR-1275 to promote ILK expression. **a** Predicted binding sites for miR-1275 in the 3′UTR of ILK or MIR143HG from the miRBase database. **b**, **c** The competing binding relationships among MIR143HG, miR-1275 and ILK were verified by dual-luciferase reporter assay. ^*^P < 0.05

Bioinformatic analysis of the binding sites in the lncRNA MIR143HG, miR-1275, and ILK on the miRBase database revealed conserved miR-1275 binding sites in lncRNA-MIR143HG and the 3′ UTR of ILK (Fig. 4a). Dual-luciferase reporter assays of the association between miR-1275 and MIR143HG or ILK revealed that miR-1275 mimics suppressed pmirGLO-MIR143HG luciferase activity but not that of the pmirGLO-MIR143HG mutant (Fig. 4b). Remarkably, miR-1275 mimics dampened luciferase activity in cells transfected with pmirGLO-ILK but not the pmirGLO-ILK mutant (Fig. 4c), suggesting that miR-1275 may induce post-transcriptional silencing of its target genes by binding to the 3′ UTR of ILK mRNA or to specific sites on the lncRNA MIR143HG. Luciferase activity of ILK wild-type reporters was significantly elevated by over-expressing MIR143HG but not MIR143HG mutant, while co-transfection with miR-1275 mimics abolished this effect (Fig. 4c), implying MIR143HG-mediated sequestration of miR-1275 as the cause of ILK up-regulation. Collectively, these data indicated that MIR143HG may modulate ILK expression by acting as a ceRNA for miR-1275.

### β-Elemene suppressed the ILK/Akt pathway and cell viability via the MIR143HG/miR-1275/ILK axis

**Fig. 5:**
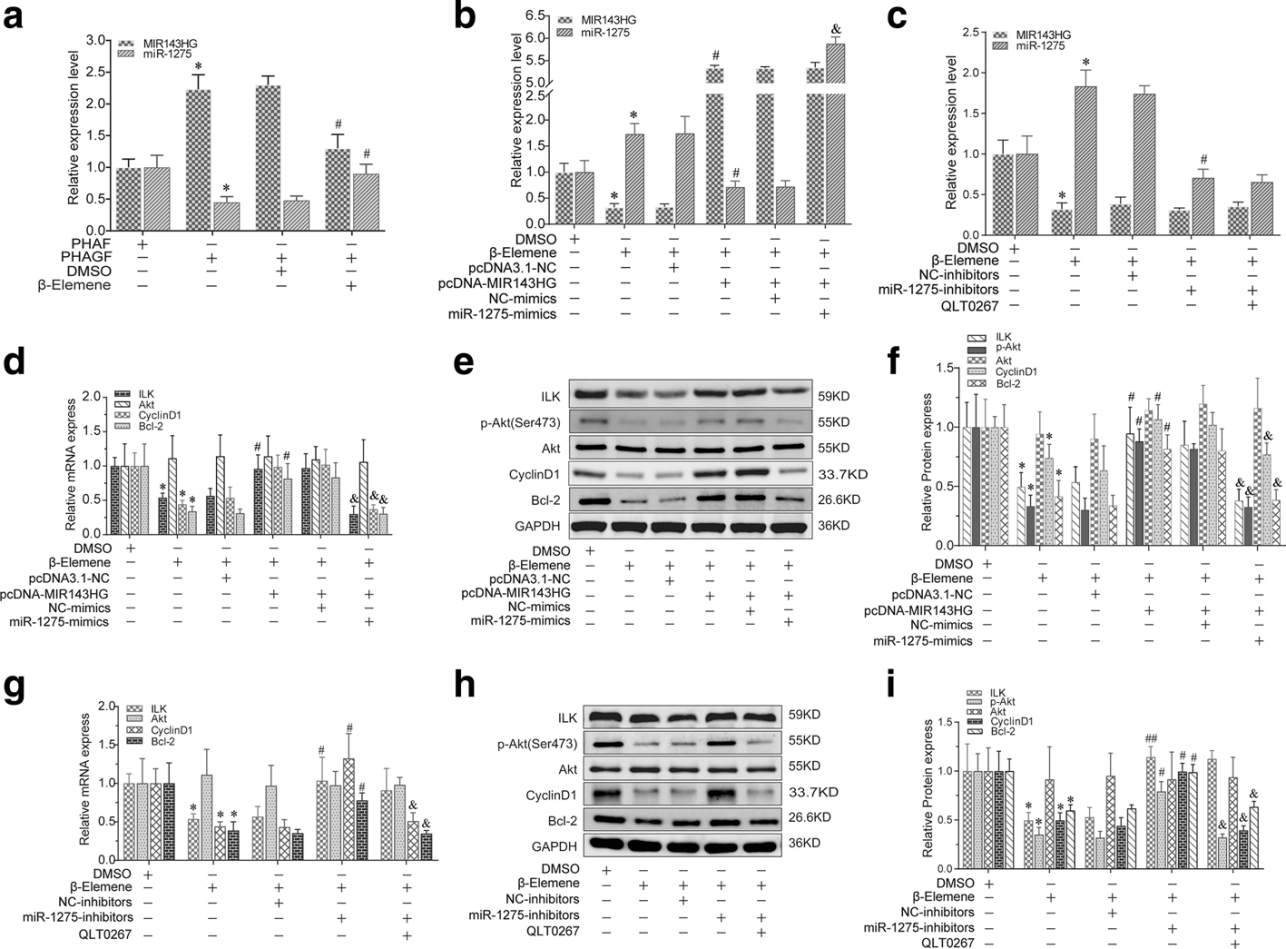
β-Elemene suppressed ILK/Akt pathway by affecting the MIR143HG/miR-1275/ILK axis in PHAGF. **a** The expression level of MIR143HG and miR-1275 was analyzed by RT-qPCR in PHAF, PHAGF, and PHAGF treated with β-elemene. **b**, **c** RT-qPCR confirmed the transfection efficiency of pcDNA3.1-MIR143HG, miR-1275 mimics or miR-1275 inhibitors. **d**, **g** RT-qPCR was used to detect the ILK/Akt pathway-related mRNAs in PHAGF under indicated treatment. **e**–**f**, **h**–**i** Western blotting was used to detect expression of ILK/Akt pathway-related proteins in PHAGF under indicated treatment. ^*^P < 0.05, ^#^*P* < 0.05, ^##^*P* < 0.01, ^&^*P* < 0.05, versus solvent control group (DMSO) or NC group (negative control)

**Fig. 6:**
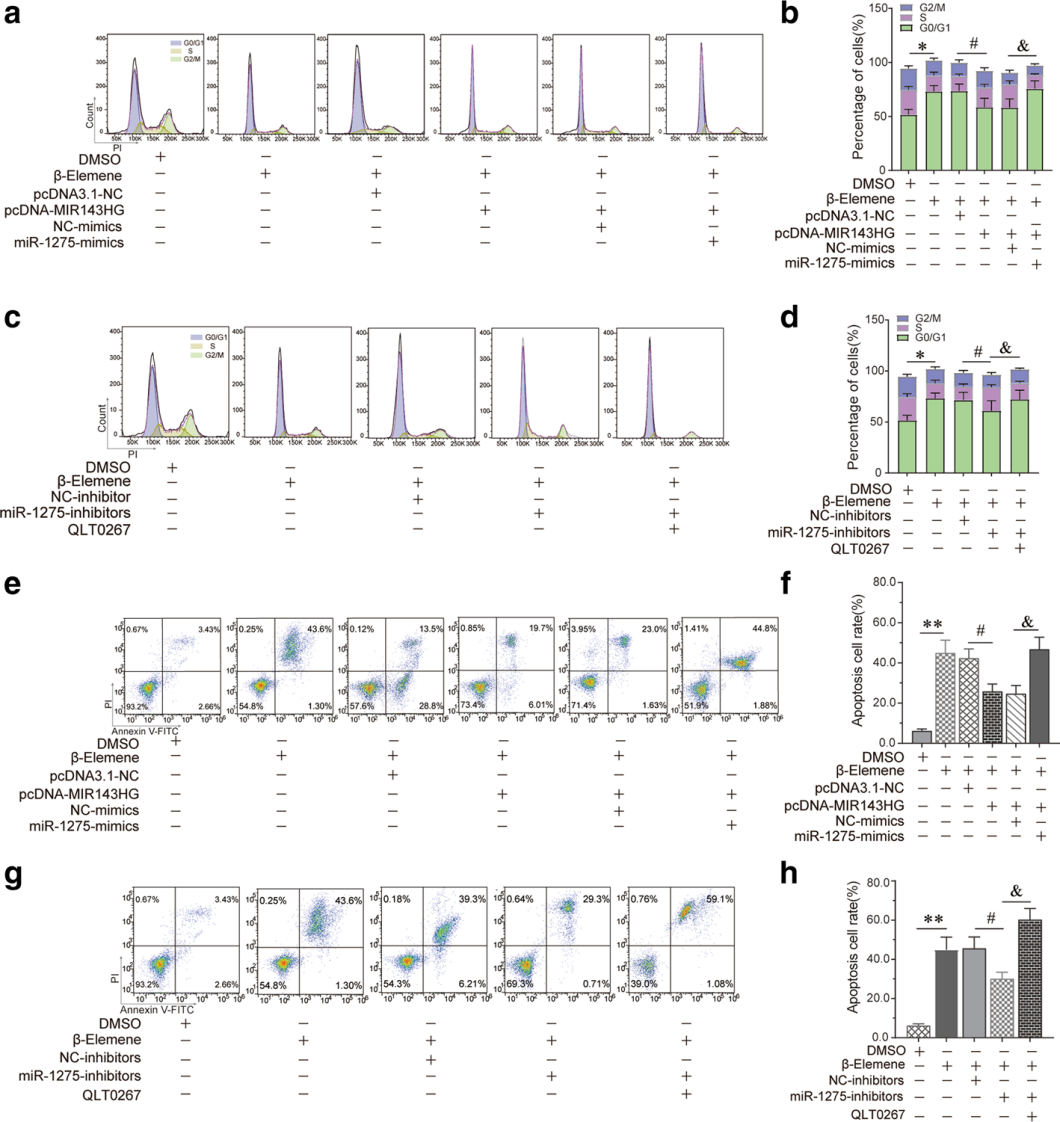
β-Elemene induced PHAGF G0/G1-phase arrest and apoptosis via MIR143HG/miR-1275/ILK axis. **a**–**d** Cell cycle distributions in PHAF, PHAGF and PHAGF under indicated treatments were analyzed by flow cytometry, and their corresponding statistical graphs were presented (comparing the rate of cell-cycle arrest in G0/G1-phase). **e**–**h** Apoptosis in PHAF, PHAGF and PHAGF under indicated treatments was analyzed by flow cytometry, and their corresponding statistical graphs were presented (comparing the relative amount of early apoptosis plus late apoptosis). ^*^P < 0.05; ^**^P < 0.01; ^#^*P* < 0.05; ^##^*P* < 0.01; ^&^*P* < 0.05

**Fig. 7:**
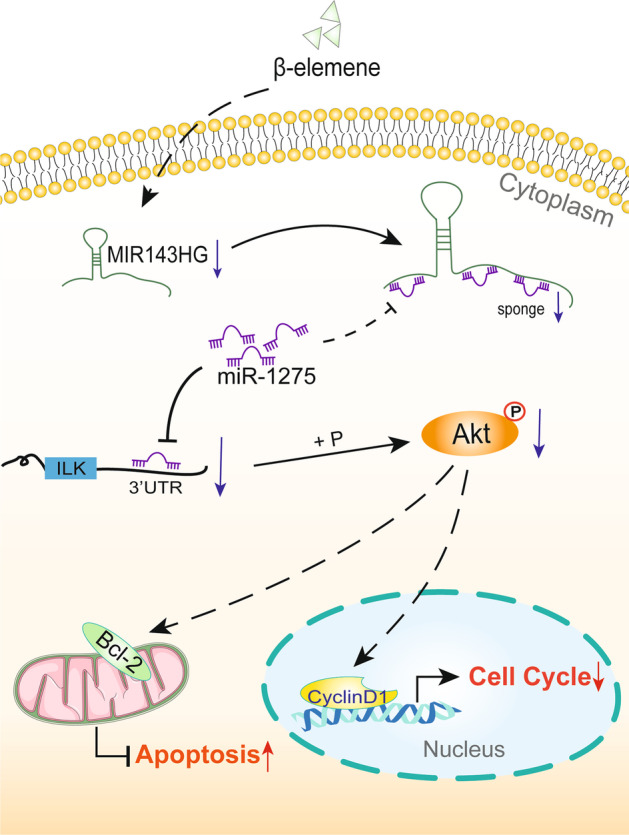
Schematic diagram of the proposed mechanism by which β-elemene regulates the cell cycle and apoptosis in PHAGF: Both MIR143HG and ILK may act as a ceRNA to sponge miR-1275. MIR143HG/miR-1275/ILK axis mediates the effect of β-elemene on PHAGF cell cycle and apoptosis by modulating the ILK/Akt pathway

Relative to PHAF, RT-qPCR analysis of MIR143HG and miR-1275 expression revealed notable MIR143HG up-regulation and marked miR-1275 down-regulation in PHAGF, and this dysregulated expression could be restored to normal after β-elemene intervention (Fig. 5a). To validate the regulatory relationship on the MIR143HG/miR-1275/ILK axis in β-elemene-treated PHAGF and its role in β-elemene's effect on PHAGF, we first confirmed the transfection efficiency of pcDNA3.1-MIR143HG, miR-1275 mimics or miR-1275 inhibitors in β-elemene treated PHAGF by RT-qPCR-detection of MIR143HG and miR-1275 expression (Fig. 5b, c). Afterwards, we explored the effects of the MIR143HG/miR-1275/ILK axis on the ILK/Akt pathway, the PHAGF cell cycle and apoptosis. RT-qPCR and Western blotting showed that pcDNA3.1-MIR143HG transfection attenuated β-elemene effects on the expression or activity of ILK, Akt, cyclin D1 and Bcl-2, while co-transfection with miR-1275 mimics reversed pcDNA3.1-MIR143HG’s effect (Fig. 5d–f). Transfection of miR-1275 inhibitors also attenuated β-elemene's effects on these genes, while treatment with QLT0267 eliminated the effect of miR-1275 inhibitors (Fig. 5g–i). Flow cytometry analysis showed that transfection with pcDNA3.1-MIR143HG or miR-1275 inhibitors reduced β-elemene-triggered G0/G1 phase arrested PHAGF and apoptotic PHAGF (Fig. 6). Additionally, co-transfection of miR-1275 mimics with pcDNA3.1-MIR143HG, or transfection of miR-1275 inhibitors plus QLT0267 treatment, again increased the proportion of G0/G1 phase arrested PHAGF and apoptotic PHAGF (Fig. 6). Overall, these results suggest β-elemene represses the ILK/Akt signaling pathway and induces PHAGF G0/G1 cell cycle arrest and apoptosis via the MIR143HG/miR-1275/ILK axis, as presented schematically in Fig. 7.

### β-Elemene promoted the regression of airway granulation tissue and alleviated tracheal stenosis

**Fig. 8:**
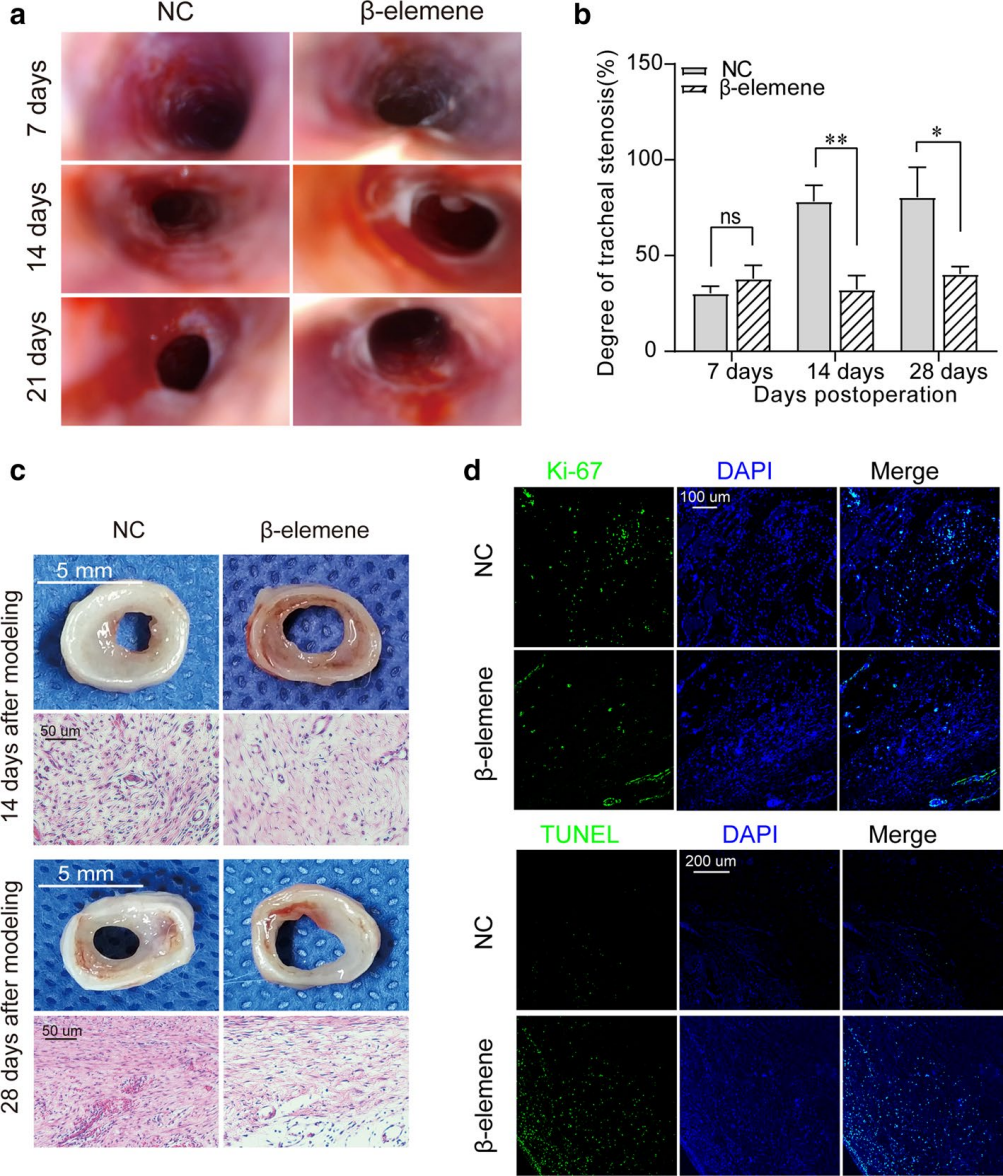
β-Elemene prevented tracheal stenosis in a rabbit model of tracheal stenosis. **a**, **b** Endoscopic examination and measurement of tracheal stenosis in rabbit models treated with β-elemene; ^*^P < 0.05; ^**^P < 0.01; ^#^*P* < 0.05; ^##^*P* < 0.01; ns, not significant. **c** Gross examination and H&E staining of the stenotic trachea. **d** Immunofluorescent staining of Ki67. **e** Representative images of TUNEL staining

In order to clarify the therapeutic efficacy of β-elemene and further verify its mechanism of action, we conducted an in vivo study based on our modified rabbit model of tracheal stenosis. As shown in Fig. 8a, b, endoscopic imaging showed that on the 7th day after modeling, the model rabbits entered the granulation hyperplasia stage, and the proliferated granulation tissue in the airway caused lumen stenosis. The stenosis rates of the two groups were 30.3 ± 3.7% and 38.0 ± 6.9%, respectively, without a significant difference between the two groups. One week after local endotracheal injection (14th day after modeling), granulation tissue continued to proliferate to a peak in the control group, severe lumen stenosis occurred, and the stenosis rate reached 78.3 ± 8.4%; however, the granulation tissue proliferation in the β-elemene group was inhibited or even subsided, and the stenosis rate of the lumen in the β-elemene intervention group was 32.3 ± 7.3%, which was significantly lower than that in the NC group. When the model rabbits entered the scar stage (28 days after modeling), scar contracture was obvious in the control group, with a stenosis rate of 80.5 ± 15.7%, while the β-elemene group had less scarring and only slight stenosis, with a stenosis rate of 40.4 ± 3.9%, which was significantly lower than that in the NC group.

### β-Elemene inhibited proliferation of airway granulation fibroblasts and promoted their apoptosis in vivo

As gross examination and H&E staining showed (Fig. 8c), 1 week after drug intervention (14th day after modeling), there was highly proliferative granulation tissue causing stenosis in the tracheal lumen in the NC group, and microscopically, the fibroblasts in the granulation tissue proliferated explosively and arranged disorderly, while the granulation tissue thickness of the β-elemene treatment group was significantly lower than that of the NC group. Microscopy showed that the interstitial edema had subsided, the number of fibroblasts was reduced, and the nuclei were atrophied in the granulation. Moreover, immunofluorescent staining of Ki67, which is present during the active phases of the cell cycle, showed that the expression of Ki67 in fibroblasts in the β-elemene group was significantly lower than that in the NC group (Fig. 8d). Meanwhile, in situ apoptosis detection by TUNEL staining showed that the apoptosis fluorescence of granulation fibroblasts in the β-elemene group was significantly higher than that in the control group (Fig. 8e). All the above evidence indicates that the proliferation of airway granulation fibroblasts was inhibited and the apoptosis was significantly promoted in the β-elemene group. At the scar maturation stage (28 days after modeling), the fibroblasts in the stenosis tissue transformed into mature fibroblasts, the control group developed significant annular scar contracture stenosis with rich collagen deposition, while the β-elemene intervention group had only mild luminal scar stenosis with a sparse collagen component (Fig. 8c). These results suggest that β-elemene inhibits the proliferation of airway granulation tissue fibroblasts and promotes its apoptosis, thereby attenuating granulation tissue hyperplasia, and ultimately alleviating airway scar stenosis.

### β-Elemene affected the MIR143HG/miR-1275/ILK axis and ILK/Akt pathway in vivo

**Fig. 9:**
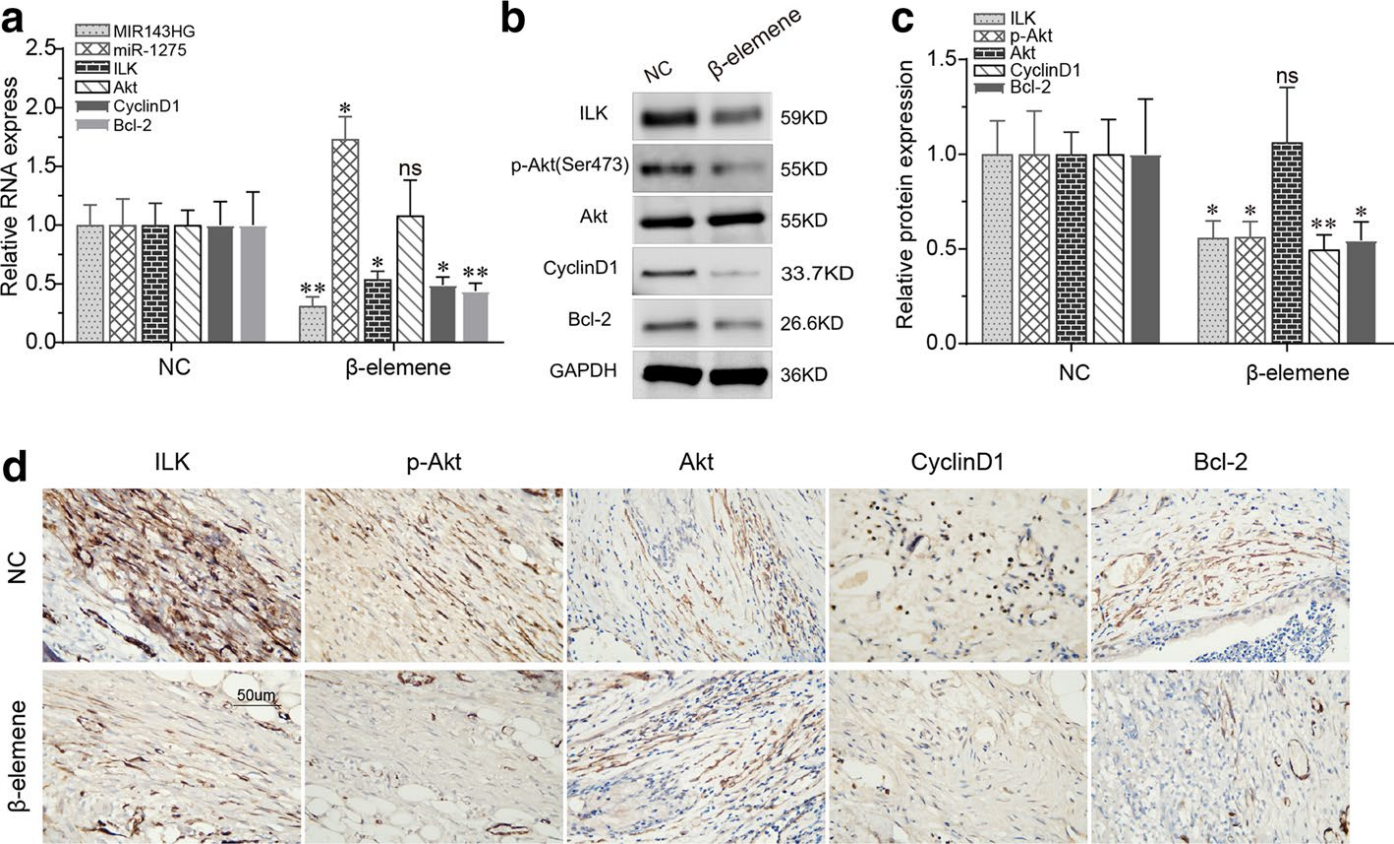
β-Elemene affected MIR143HG/miR-1275/ILK axis and ILK/Akt pathway in vivo. **a**–**c** RT-qPCR and Western blotting were used to detect activity of the MIR143HG/miR-1275/ILK axis and ILK/Akt pathway in rabbit models treated with β-elemene. ^*^P < 0.05, ^**^P < 0.01; *ns* not significant. **d** Representative images of immunohistochemistry staining of ILK/Akt pathway-related proteins

Furthermore, the MIR143HG/miR1275/ILK axis and ILK/Akt pathway were analyzed in the animal tracheal specimens. RT-qPCR analysis showed that compared with the NC group, the expression levels of miR143HG, ILK, cyclin D1 and Bcl-2 in the β-elemene group were significantly down-regulated while the expression of miR-1275 was up-regulated (Fig. 9a). Moreover, Western blotting analysis showed that the differential expression of ILK, Bcl-2 and cyclin D1 in the two groups was consistent with the results of RT-qPCR, and at the same time, activated Akt (p-Akt) protein was also significantly down-regulated in the β-elemene group (Fig. 9b, c). These findings were further verified by immunohistochemical staining (Fig. 9d), which showed that the expression levels of ILK, p-Akt, Bcl-2 and cyclin D1 were significantly reduced in the β-elemene group. Apparently, the effects of β-elemene on the ceRNA axis and ILK/Akt pathway in airway granulation tissue were consistent with the findings demonstrated in vitro, further supporting the mechanism of β-elemene's action (Additional file 1).

## Discussion

To prevent benign airway restenosis, there is an urgent need to identify effective medications against abnormal airway granulation fibroblast activity, which plays a critical role in granulation tissue hyperplasia and scar formation during wound healing after endobronchial intervention in the human airway. β-Elemene, as a widely used effective ingredient of traditional Chinese medicine, has been positively evaluated in our previous studies for its potential efficacy in the prevention and treatment of benign airway restenosis [14, 27]. Here, we sought to further elucidate its underlying molecular mechanism, and further verify its efficacy and mechanism in vivo. To this end, we first used microarray analysis to identify lncRNA and mRNA expression changes in PHAGF after β-elemene treatment, and subsequent bioinformatics and RT-PCR analyses confirmed that ILK and its downstream genes (cyclin D1 and Bcl-2) in the ILK/Akt signaling pathway as well as lncRNA MIR143HG, which could act as a ceRNA against ILK binding to miR-1275, were substantially down-regulated.

ILK (integrin-linked kinase) is a hub gene in the focal adhesion signaling pathway and an upstream gene in many sub-pathways, including ILK/Akt. ILK phosphorylates and activates Akt at Ser473, a key modulator of various cellular functions including apoptosis and cell cycle progression. Akt activates cyclin D1, which is a key regulator of cell cycle progression in the G1/S phase, and inhibition of its expression can lead to cell arrest in the G1 phase, thereby modulating mitogenesis and proliferation [36, 37]. Akt also phosphorylates and inactivates GSK-3 (Ser9), thus suppressing its inhibition of downstream substrate Bcl-2, a pro-survival factor that inhibits caspase-9 cascade activation in the mitochondrial apoptosis pathway, thus suppressing apoptosis and promoting cell survival [38–40]. Studies have shown that ILK is widely involved in impaired tissue repair, include nerve fibers, myocardium, and hair follicles, and plays an important regulatory role in the processes of renal interstitial fibrosis, pulmonary fibrosis and liver fibrosis [41–43]. Here, we found that expression or activity of key ILK/Akt pathway factors (ILK, Akt, cyclin D1 and Bcl-2) is markedly enhanced in PHAGF relative to PHAF, and that β-elemene induces PHAGF G0/G1 arrest and apoptosis by suppressing these relevant effector genes in ILK/Akt signaling. It is conceivable that the ILK/Akt pathway may regulate proliferation and survival of various types of abnormal fibroblasts and may be promising for prevention and treatment of fibrosis. Abnormal activation of fibroblasts in wounds has been reported to lead to the occurrence of pathological repair [44, 45]. This perspective is supported by our data, as we found that compared with normal airway fibroblasts, the ILK/Akt pathway was abnormally activated in PHAGF, leading to stronger proliferative activity and anti-apoptotic ability, and β-elemene suppressed the viability of PHAGF by attenuating its ILK/Akt pathway.

MiRNAs can specifically bind to complementary sequences (usually the binding site is located in the 3ʹ UTR region of a gene) called miRNA response elements (MREs) through their "seed regions", resulting in the inhibition or degradation of RNA containing these complementary sequences. This mechanism of targeted post-transcriptional regulation has been widely recognized [46, 47]. Competing endogenous RNA (ceRNA), a novel mechanism for the interregulation of RNAs (including lncRNA, and mRNA) that competitively bind the same MREs, have been shown to modulate various complex and subtle biological processes, including embryonic development and pathogenesis of various disorders, including cancer [17, 48, 49]. More and more studies have reported the important roles of this regulatory mechanism in tumorigenesis and tumor development [50]. Lin et al. [51] reported that lncRNA MIR143HG competitively binds to miR-155, which inhibits APC expression in Wnt signaling, thereby suppressing hepatocellular carcinoma cell proliferation and metastasis. Xie et al. [34] reported that the MIR143HG/miR‐1275/AXIN2 axis modulates bladder cancer development by influencing Wnt/β‐catenin signaling. lncRNA FAM225A has been shown to act as a ceRNA absorbing miR-1275, up-regulating ITGB3 and promoting metastasis in nasopharyngeal carcinoma [52]. LncRNA Hand2-AS1 competitively binds to miR-1275, targeting KLF14 to inhibit rectal cancer progression [53].

However, little attention has been paid to the role of ceRNA regulatory networks in regulating the function of fibroblasts in hyperplastic granulation, or their potential as drug targets in these cells. The molecular basis of β-elemene mediated suppression of human airway granulation fibroblasts via ceRNA modulation has hardly been reported before. Here, we first identified MIR143HG and -1275 as being dysregulated in PHAGF relative to PHAF, and that β-elemene can reverse their expression. Using dual-luciferase reporter assays, we confirmed that miR-1275 has a target relationship with MIR143HG and ILK, and MIR143HG competed with ILK for miR-1275 binding, indicating that MIR143HG and miR-1275 are each other's ceRNA. Furthermore, we demonstrated through a series of loss-of-function and gain-of-function experiments that the MIR143HG/miR-1275/ILK axis mediates β-elemene’s regulation of the ILK/Akt pathway and ultimately mediates the promotion of β-elemene on the cell phenotype (cell cycle arrest and apoptosis) corresponding to the downstream effector genes (cyclin D1 and Bcl-2) of the ILK/Akt pathway.

Furthermore, the in vivo study clearly revealed that β-elemene could inhibit the proliferation of fibroblasts in granulation tissue and promote its apoptosis, and with the decrease of granulation fibroblasts, granulation hyperplasia weakened or even atrophied, thus forming less contracture scar tissue in the scar maturation stage and alleviating airway stenosis. Moreover, the present study confirmed that the effects of β-elemene on the ceRNA axis and ILK/Akt pathway activity in airway granulation tissue were in line with our in vitro data, which further supports that the MIR143HG/miR-1275/ILK axis modulating the ILK/Akt pathway plays a critical role in β-elemene's preventive and therapeutic effects on benign airway stenosis.

However, from a preliminary perspective of microarray analysis, β-elemene affected the expression of multiple mRNAs and lncRNAs. Thus, whether it functions via additional mechanisms through multiple targets warrants further investigation. Additionally, the molecular basis of β-elemene’s regulation of MIR143HG requires further interrogation. Inflammatory cytokines and angiogenic cytokines are crucial mediators of tissue repair. β-elemene has been reported to suppress the release of pro-inflammatory factors by neutrophils and macrophages [54, 55], and to inhibit melanoma growth and metastasis via VEGF-mediated angiogenesis [56]. Whether the anti-inflammatory and anti-angiogenic effects of β-elemene influence its inhibitory effects on airway granulation tissue needs to be further explored.

## Conclusions

In summary, the current study demonstrates that MIR143HG and ILK may act as ceRNA to sponge miR-1275, and the MIR143HG/miR-1275/ILK axis mediates β-elemene-induced PHAGF cell cycle arrest and apoptosis by modulating the ILK/Akt pathway, thereby inhibiting airway granulation hyperplasia and alleviating airway stenosis. These findings provide further insight into the regulatory mechanism of β-elemene involving ncRNAs and provide experimental evidence supporting the clinical application of β-elemene in tracheal stenosis.

## Supplementary Information

**Additional file 1. ** Supplementary Information-Original images for western blot.

## Data Availability

The datasets generated and analyzed during the current study are available in the Gene Expression Omnibus (GEO): https://www.ncbi.nlm.nih.gov/geo/query/acc.cgi?acc=GSE155297.

## Abbreviations

Bcl-2: B-cell lymphoma-2
CCK-8: Cell counting kit-8
DMEM: Dulbecco’s modified Eagle’s medium
DMSO: Dimethyl sulfoxide
EDTA: Ethylene diamine tetraacetic acid
FBS: Fetal bovine serum
FITC: Fluorescein isothiocyanate
GAPDH: Glyceraldehyde-3-phosphate dehydrogenase
HRP: Horseradish peroxidase
ILK: Integrin-linked kinase
IHC: Immunohistochemistry
KEGG: Kyoto Encyclopedia of Genes and Genomes
OD: Optical density
PBS: Phosphate-buffered saline
PMSF: Phenylmethylsulphonyl fluoride
PVDF: Polyvinylidene fluoride
RIPA: Radio immunoprecipitation assay
RT-qPCR: Quantitative real-time reverse transcriptase–polymerase chain reaction
SD: Standard deviation
SDS-PAGE: Sodium dodecyl sulfate–polyacrylamide gel electrophoresis
TBST: Tris buffered saline Tween
