# Supplementary material for: β-elemene alleviates airway stenosis via the ILK/Akt pathway modulated by MIR143HG sponging miR-1275
Source: Cell Mol Biol Lett. 2021 Jun 12;26:28. doi: 10.1186/s11658-021-00261-0 (PMC8199800; doi:10.1186/s11658-021-00261-0)

Supplementary Information

β-Elemene Alleviate Post-traumatic Airway Stenosis via ILK/Akt Pathway Modulated by MIR143HG Sponging miR- 1275

Supplementary Information: The original, unprocessed versions of immunoblot membranes in Fig. 2, Fig.5 and Fig. 9.

Figure 2

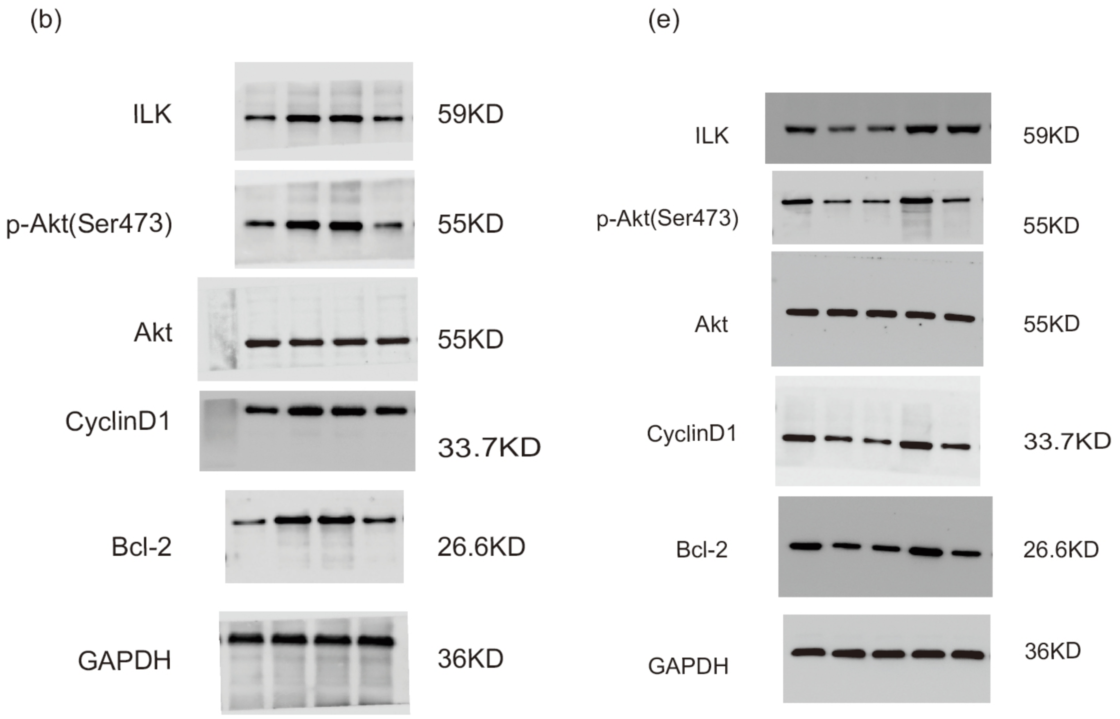

Figure 5

(e)

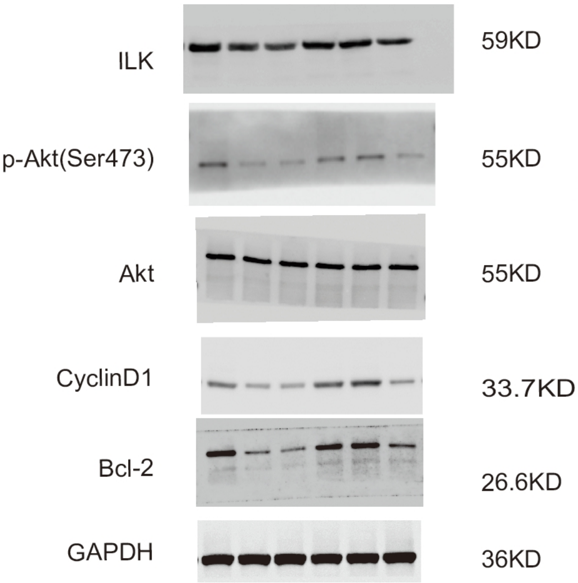

(h)

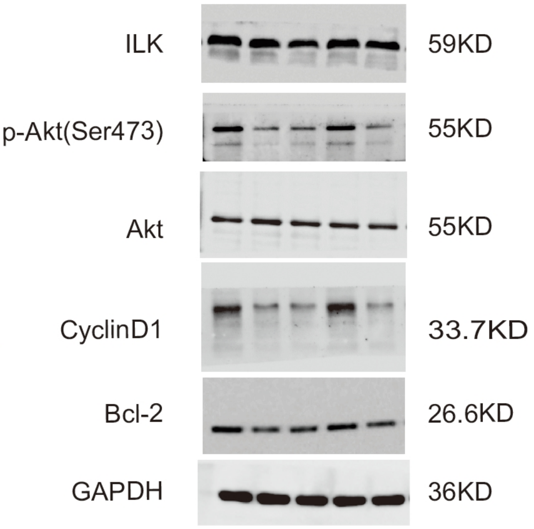

Figure 9

(b)

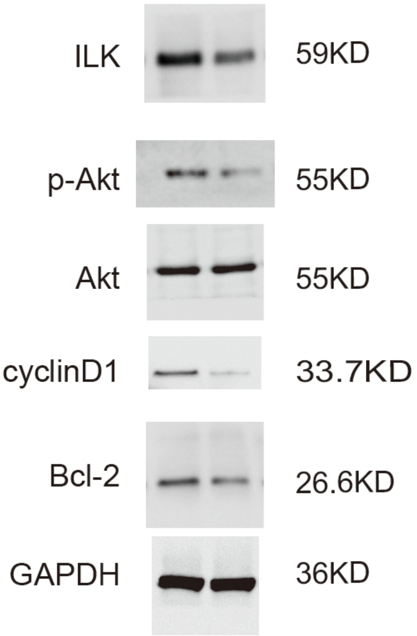

Supplement: Supplementary file 1 — Additional file 1. Supplementary Information-Original images for western blot. [file 11658_2021_261_MOESM1_ESM.pdf]
